# Peptide antibody tools to dissect specific functions of eIF4A paralogs in cancer

**DOI:** 10.1007/s12672-025-04155-x

**Published:** 2025-11-26

**Authors:** Shobhit Srivastava, Azeezat Osikoya, David Terrero, Dayanidhi Raman

**Affiliations:** Department of Cell and Cancer Biology, College of Medicine and Life Sciences, University of Toledo Health Science Campus, 3000 Arlington Avenue, MS 1010, Toledo, OH 43614 USA

**Keywords:** mRNA translation, eIF4A1, eIF4A2, Specific peptide antibody, Triple-negative breast cancer

## Abstract

Eukaryotic translation initiation factors (eIFs) are key regulators of messenger RNA (mRNA) translation, a process highly dysregulated in cancer. Among these paralogs, eIF4A1 and eIF4A2 are closely related DEAD box mRNA helicases that, despite their high sequence identity, play distinct roles in cellular homeostasis and tumorigenesis. In the context of triple-negative breast cancer (TNBC), where dysregulated protein translation drives tumor progression and metastasis, high rate of relapse, and resistance to radio- and chemotherapy, an understanding of the unique contributions of eukaryotic initiation factor 4 A (eIF4A) paralogs is important. Current commercial antibodies are not validated to claim the specificity towards these helicases. To address this, we developed and validated paralog-specific antibodies to unequivocally detect eIF4A1 and eIF4A2 by leveraging unique sequence differences in their N-terminal regions. Using affinity-purified, rabbit peptide-antibody generation followed by validation through immunoblot analyses of knockout and knockdown cellular lysates, we confirmed the high specificity of the antibodies and the absence of any cross-reactivity. These results establish that these antibodies are invaluable tools for dissecting the distinct roles of eIF4A1 and eIF4A2 in translational control and potential therapeutic strategies targeting these factors to overcome chemoresistance, primary tumor progression, and metastasis. This study provides a foundation for future investigations into the functional divergence of eIF4A paralogs, their functional ramifications in cancer, and their implications in tumor progression and metastasis.

**Supplementary Information:**

The online version contains supplementary material available at 10.1007/s12672-025-04155-x.

## Introduction

Initiation of messenger RNA (mRNA) translation is a tightly regulated process and, importantly, a rate-limiting step in cap-dependent translation. In addition, it is a major driver of primary tumor progression and metastasis [1]. The eukaryotic translation initiation factor 4 F (eIF4F) complex plays a vital role in this process. The cap-binding eIF4F complex bound to the 5’-leader region of the mRNA recruits the pre-initiation complex, 43 S ribosome, to form the 48 S translation initiation complex. The directional scanning of the mRNA for any secondary structures in the 5’-leader region or 5’-untranslated region (5’-UTR) occurs until it locates the start AUG codon under proper context [2, 3]. The eIF4F heterotrimeric complex is comprised of eIF4E, a cap-binding protein; eIF4A1, a DEAD (Aspartate-Glutamate-Alanine-Aspartate)-box mRNA helicase; and eIF4G, a big scaffold protein that positions eIF4E and eIF4A1 appropriately so that the ancillary factors eIF4B or eIF4H (mutually exclusive) can get recruited and increase the translation efficiency [4]. As the name indicates, the family of DEAD-box mRNA helicases have a conserved “DEAD” motif in their catalytic core [5, 6]. These enzymes play essential roles in unwinding stem-loop structures (SLS) or secondary structures at the 5’-leader regions [7, 8]. In doing so, the eIF4F complex promotes the expression of many hallmarks of cancer such as sustained proliferative signaling, induction of chemoresistance, resistance of cell death, activation of immune checkpoint, and activation of migration, invasion and metastasis.

In the DEAD-box family of helicases, there are three paralogs, eIF4A1 (DDX2A), eIF4A2 (DDX2B), and eIF4A3 (DDX48), of which eIF4A1 and eIF4A2 share ~ 90% identity at the amino acid level while eIF4A3 is slightly divergent [9]. The gene for eIF4A1 is located on the human chromosome 17q13, while for eIF4A2 it is located on 3q28. eIF4A1 is the most abundant paralog expressed in a wide variety of tissues, and is involved in multiple cellular processes, such as the regulation of gene expression, cell cycle control, and stress responses [10, 11]. Importantly, eIF4A1 functions as an ATP-dependent processive mRNA helicase for translation of many vital oncogenic mRNAs such as c-MYC, BIRC5 or survivin, Mdm2, rho kinase 1 (ROCK1), cyclin D1, and cyclin D3 [12, 13]. Collectively, these proteins are critical for tumor cell fitness and survival, proliferation, metastasis, and chemoresistance. Enhanced total protein levels and activity of eIF4A1 correlate with poor prognosis and drug resistance in various cancers, including TNBC [14]. Dysregulated expression of eIF4A1 has been implicated in several cancers, including breast cancer [14], melanoma [15], prostate cancer [16, 17], and pancreatic ductal adenocarcinoma (PDAC) [18].

In TNBC, where the lack of efficacious targeted therapies contributes to a high rate of relapse, increased metastases, and mortality, aberrant expression of eIF4A1 has been reported to drive the translation of oncogenic mRNAs involved in cancer-related pathways, such as those regulating cell proliferation and survival (cyclins D1/D3, survivin), pluripotency (SOX2, OCT4, NANOG), and chemoresistance [ATP-binding cassette (ABC) drug efflux transporters (ABCB1, ABCC1, and ABCG2), which efflux neoadjuvant chemotherapeutic drugs (NACT) contributing to multidrug resistance] [19, 20]. Additionally, eIF4A1 supports the translation of transcription factors involved in immune suppression, facilitating tumor evasion from immune surveillance [21, 22].

Both eIF4A1 and eIF4A2 can associate with the eIF4F complex, but eIF4A1 being more abundant, it is predominantly recruited to the eIF4F complex and has been extensively studied [23]. Though eIF4A1 and eIF4A2 are interchangeable, the downregulation of the functionality of eIF4A1 is not rescued by eIF4A2 [9]. Interestingly, total or functional loss of eIF4A1 has been observed to result in a substantial increase in eIF4A2 expression at the mRNA and the protein level [24, 25]. Taken together, this shows that eIF4A1 and eIF4A2 have dissimilar functionalities but may also have some overlapping functions.

Traditionally, eIF4A2 has been reported to play an important role in microRNA (miRNA) repression by preferentially binding to CNOT7 (CCR4-NOT transcription complex subunit 7), a member of the CCR4-NOT complex [26]. Recently, eIF4A2 has been demonstrated to mediate the translation of Ddx6 mRNA. The Ddx6 helicase and eIF4A2 then act in concert to repress the Zscan4 translation that inhibits the development of embryonic stem cells (ESC) [27]. Unlike eIF4A1, whose genetic ablation has been implicated in hampering the translation of oncogenic mRNAs [19], depletion of eIF4A2 may only have a negligible effect, as it is currently known to affect the translation of only a few mRNAs. In addition to translating DDX6, eIF4A2 may have novel oncogenic functions, but the novel downstream effectors of eIF4A2 are presently unknown. Importantly, when eIF4A1 is genetically ablated or pharmacologically targeted, there is an enhanced expression of eIF4A2 at the mRNA and the protein level. Still, the functional outcome is not clear since eIF4A2 by itself is not able to functionally rescue either eIF4A1-knock out or eIF4A1i [28]. To address these, we developed paralog-specific, affinity-purified rabbit peptide antibodies to investigate the distinct contributions of eIF4A1 and eIF4A2 in cancer biology. These antibodies provide a foundation for future studies on translational regulation and could facilitate the development of novel paralog-specific therapeutic strategies for precision medicine-based approach in the cancer clinic.

## Materials and methods

### Recombinant proteins

Purified recombinant proteins, eIF4A1 (cat.TP303298) and eIF4A2 (cat.TP305623), were purchased from Origene Tech., Rockville, MD, USA.

### Commercial antibodies

To evaluate the specificity of commercially available antibodies, we tested their cross-reactivity using purified recombinant eIF4A1 and eIF4A2 proteins. The antibodies assessed include eIF4A1 antibody from Abcam (ab31217, Waltham, MA, USA,) [29], Cell Signaling Tech.(#2940, Danvers, MA, USA), Origene Tech.(TA302076, Rockville, MD, USA) and Thermo Scientific (#711505, Carlsbad, CA, USA), while eIF4A2 antibody was purchased from Santa Cruz Biotechnology (sc-137148, Dallas, TX, USA) [26], Origene Tech. (TA345787, Rockville, MD, USA,) and Abcam (ab31218, Waltham, MA, USA).

### Protein sequence alignment

To identify any non-overlapping peptide regions as antigens for raising rabbit antibodies, we aligned amino acid sequences of eIF4A1 and eIF4A2 online using uniport ID: P60842 and Q14240 respectively via LALIGN program.

### Peptide synthesis and antibody production

Through an alignment of the protein primary sequences, the disparate region was mainly found at the N-terminal regions of eIF4A1 and eIF4A2. The epitope peptide was commercially synthesized, and antibodies were raised in rabbits against eIF4A1 and eIF4A2 commercially by GenScript (Piscataway, NJ, USA). 5 mg of custom epitope peptides corresponding to the differential N-terminal sequences of eIF4A1 (ASQDSRSRDNC) and eIF4A2 (GSADYNREHGC) were synthesized. A cysteine residue was added at the C-terminus to enable conjugation to carrier protein. These were subsequently conjugated to the carrier protein, keyhole limpet hemocyanin (KLH) to enhance the antigenicity of the epitope peptides. The conjugated peptides were used to generate polyclonal anti-peptide antibodies against eIF4A1 and eIF4A2 paralogs in rabbits following standard protocols [30]. After an initial injection to initiate the antibody production, a booster was administered. The N-terminal peptide from eIF4A2 produced a robust immune response and the antibody titer was tested by an indirect ELISA. However, initially the N-terminal peptide from eIF4A1 did not elicit an optimal antibody titer and was boosted again with the antigenic peptide.

### Cell culture

The human TNBC cell line MDA-Bone-Un (MDA-MB-231 re-isolated from mouse bone metastatic lesions) was originally obtained from Dr. Julie A. Rhoades (Sterling) from the Vanderbilt Center for Bone Biology, Vanderbilt University Medical Center, Nashville, TN. MDA-Bone-Un cells typically metastasize to the lungs when orthotopically implanted into 4th mammary fat pads of 8 wk-old female mice. The eIF4A1-KO cells were generated previously by a CRISPR-Cas9 approach [19] and doxycycline-induced eIF4A2 knockdown is produced in this study in MDA-Bone-Un cell line.

### Doxycycline-induced knockdown of EIF4A2

To generate the doxycycline-induced *EIF4A2* knockdown, we selected two shERWOOD UltramiR Lentiviral Inducible shRNAs (ULTRA-3235034 and ULTRA-3235036) that target the *EIF4A2* coding sequence. These shRNAs are engineered into the lentiviral vector pZIP-TREG3G-ZsGreen-Puro (TLHSU2333, Transomics, Huntsville, Alabama) with a doxycycline-inducible cassette encoding ZsGreen plus a short hairpin RNA followed by TET-on-3G and puromycin resistance gene for selection of stably transfected cells. This plasmid provides everything needed for doxycycline-induced knockdown in one construct.

The doxycycline-induced knockdown of *EIF4A2* in MDA-Bone-Un cell line was accomplished by using a second-generation lentiviral packaging system [viral packaging -psPAX2 (12260, Addgene.org) and VSV-G envelope -pMD2.G (12259, Addgene.org) plasmids] transfected into immortalized human embryonic kidney cell line (HEK-293FT) [31, 32]. At 48 h and after 72 h, the pseudolentiviral particles in the cell culture supernatant were collected and concentrated using ‘Amicon Ultra-15’ centrifugal filters (Millipore-Sigma, St. Louis, MO) with 10 K nominal cut off. The concentrated lentiviral particles were transduced into MDA-Bone-Un TNBC cells. The TNBC cells harboring the lentiviral knockdown plasmid for *EIF4A2* were selected with puromycin (1 µg/mL). The inducible knockdown of *EIF4A2* in the puromycin-selected stable cell line is obtained by adding doxycycline at 1 µg/mL for 48 h.

### Immunoblot analysis

The specificity of the custom peptide- and commercial antibodies directed against eIF4A1 and eIF4A2 were validated using an immunoblotting approach. 0–20 ng of purified, recombinant eIF4A1 and eIF4A2 proteins were resolved by 10% sodium dodecyl sulfate-polyacrylamide gel electrophoresis (SDS-PAGE) and transferred onto nitrocellulose membrane (0.45 μm) overnight at constant 25 V and at 4 °C. The membranes were blocked with 5% non-fat dry milk, incubated with the purified antibodies against eIF4A1 and eIF4A2, and loading control cyclophilin B (PA1-027 A, Invitrogen, Waltham, MA) followed by horseradish peroxidase-conjugated secondary antibodies (711-035-152, Jackson ImmunoResearch, West Grove, PA, USA). Protein bands were visualized by enhanced chemiluminescence (ECL) detection (RPN2232, Marlborough, MA, Cytiva) and the emitted ECL was captured using the Syngene-G: BOX imager. The specificity of antibody was assessed by the presence of bands corresponding to the Mr range of eIF4A1 and eIF4A2 without any cross-reactivity. For the evaluation of commercial antibodies and recombinant eIF4A1 and eIF4A2 proteins, membranes were cut at the exact Mr corresponding to eIF4A1 and eIF4A2, respectively, before antibody hybridization. This was done to specifically detect the protein levels without interference from other bands. For the assessment of eIF4A1-KO cell and eIF4A2 knockdown cell lysates, membranes were also cut at Mr for the loading control, ensuring accurate normalization of protein levels.

### Immunocytochemistry (ICC)

Coverslips were pre-coated with Collagen IV (0.1 mg/mL, #C7521, Sigma–Aldrich, Saint–Loius, MO, USA)) for 2 h at RT and rinsed with 1× PBS. Cells (8 × 10⁴ cells total/coverslip) were then seeded at 50% confluence and allowed to attach and spread overnight. Following gentle washing with 1× PBS thrice, cells were fixed in 4% neutral-buffered formaldehyde (#245684, Fisher, Pittsburgh, PA) for 15 min and permeabilized with 0.2% Triton X-100 in PBS for 15 min. Cells were then blocked in 10% Donkey serum (#017-000-121, Jackson ImmunoResearch, West Grove, PA, USA) prepared in PBS for 1 h at RT. Primary peptide antibodies against eIF4A1(Rabbit 1(0.685 mg/mL); Rabbit 2(0.863 mg/mL)) and eIF4A2 Rabbit 2(1.921 mg/mL) were incubated at a final concentration of 2 µg/mL overnight at 4 °C. After washing with 1× PBS thrice, cells were incubated with ChromoTek 555 nano-secondary antibody (1:200, #srb2GCL555-1, Proteintech, Rosemont, IL, USA) for 1 h at RT. Nuclei were counterstained with Hoechst 33342 (1:10,000, #H3570; Invitrogen, Waltham, MA, USA) in the final wash at RT. Coverslips were mounted with ProLong™ Gold Antifade Mountant (Invitrogen, #P36980), and images were acquired using Leica TCS SP5 multiphoton laser scanning confocal microscope (Leica Microsystems, USA) equipped with 63× oil-immersion objective lens.

## Results

### Validation of peptide-antibodies targeted against eIF4A1 and eIF4A2

#### Production of the anti-peptide antibodies against eIF4A1 and eIF4A2

The high sequence similarity (90%) between eIF4A1 and eIF4A2 presented a challenge in trusting commercial antibodies for their specificity as they were not KO-validated. The development of paralog-specific antibodies has proven to be difficult [33]. There are no established guidelines in the fields of eIF4A1 and eIF4A2 regarding the specificities of the commercially available antibodies. Here, we present our attempt to clarify this and delineate the remarkable specificity of the purified anti-peptide rabbit antibodies against eIF4A1 and eIF4A2 from our laboratory using knockout and knockdown approaches. We also validated commercially available antibodies targeting eIF4A1 and eIf4A2. We observed a strong reactivity against the immunizing peptides as there was a robust antibody titer (immune response) against injected eIF4A1 and eIF4A2 peptides.

**Fig. 1:**
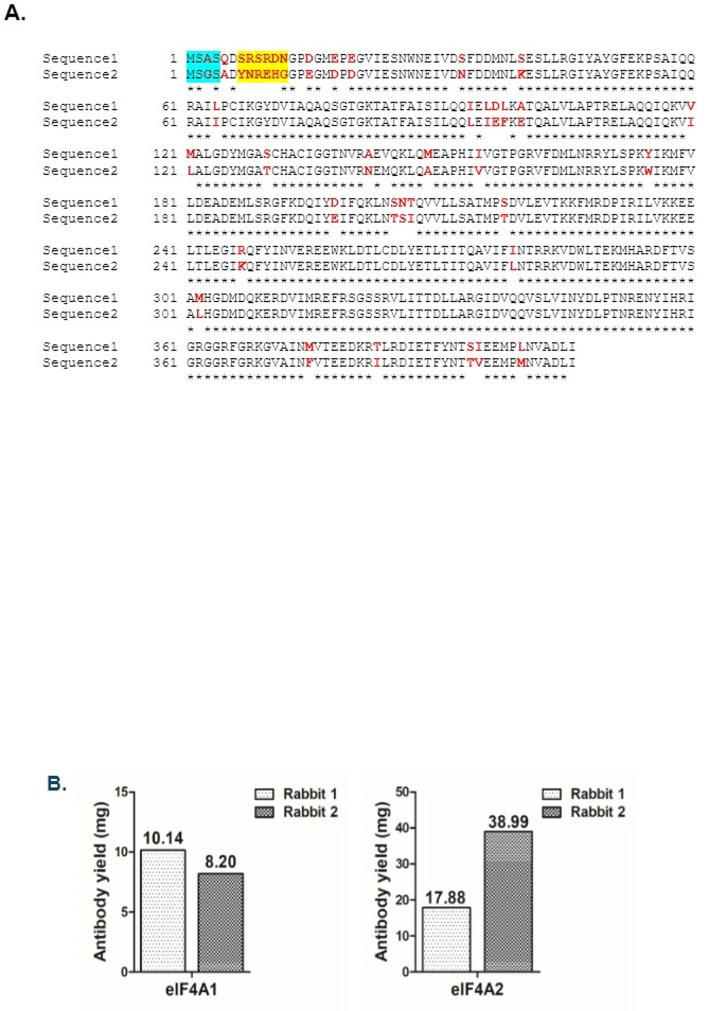
Sequence alignment of eIF4A1(P60842) and eIF4A2 (Q14240): **A** Divergent amino acid residues between eIF4A1 and eIF4A2 are highlighted in red. The sequences were aligned using the LALIGN program. Sequence alignment: 90.3% identity in 403 residues overlap; Score: 1901.0; Gap frequency: 0.0%; Sequence1: eIF4A1; Sequence2: eIF4A2. **B** Bar chart showing the total antibody yields of eIF4A1 and eIF4A2 peptide-antibodies

The N-terminal eIF4A1 epitope ‘ASQDSRSRDN’ was employed as the peptide antigen and injected into two rabbits (Fig. 1A). The sera were tested after an initial injection, and a booster was administered as the titer was not robust. The two rabbits were given a second booster and antibody titer was assessed and found to be robust (Table 1). Similarly, the N-terminal eIF4A2 epitope ‘GSADYNREHG’ was used as the peptide antigen and administered to two rabbits. The sera were similarly tested for the antibody titer, and the titer was found to be robust (Table 2). Subsequently, the antigenic peptides for eIF4A1 and eIF4A2 were covalently conjugated to Sepharose and the corresponding peptide antibodies were affinity-purified from the sera. The yield of the antibodies after immunoaffinity purification are (Fig. 1B): Anti-eIF4A1 Rabbit1 - 0.685 mg/mL (10.14 mg total), anti-eIF4A1 Rabbit2 - 0.863 mg/mL (8.2 mg total), anti- Rabbit1 - 0.988 mg/mL (17.88 mg total) and anti-eIF4A2 Rabbit2 - 1.921 mg/mL (20.30 mg total).

**Table 1:** ELISA results of the rabbit IgG control and affinity-purified antibody eIF4A1

Concentration (ng/mL)	1000.00	500.00	250.00	125.00	62.50	31.25	15.62	7.81	3.90	1.95	/	/
Dilution \Animal No.	1:1000	1:2000	1:4000	1:8000	1:16,000	1:32,000	1:64,000	1:128,000	1:256,000	1:512,000	Blank	Titer^[1]^
Rabbit 1 (0.685 mg/mL)	2.197	1.975	1.692	1.505	1.482	1.134	0.928	0.656	0.422	0.259	0.058	1:512000
Rabbit 2 (0.863 mg/mL)	2.540	2.446	2.382	2.244	1.976	1.810	1.497	1.172	0.786	0.565	0.055	> 1:512000
IgG ^[2]^	0.069	0.060	0.058	0.068	0.058	0.064	0.059	0.061	0.066	0.068	0.060	< 1:1000

^[1]^Titer is the highest dilution with Signal/Blank (S/B) >=2.1 and the OD450 of the blank is the average of two technical replicates

^[2]^ Isotype IgG control

**Table 2:** ELISA results of the rabbit IgG control and affinity-purified antibody eIF4A2

Concentration (ng/mL)	1000.00	500.00	250.00	125.00	62.50	31.25	15.62	7.81	3.90	1.95	/	/
Dilution\Animal No.	1:1000	1:2000	1:4000	1:8000	1:16,000	1:32,000	1:64,000	1:128,000	1:256,000	1:512,000	Blank	Titer^[1]^
Rabbit1(0.988mg/mL)	3.251	3.283	3.138	3.118	3.038	2.897	2.811	2.581	2.225	1.712	0.056	>1:512000
Rabbit2(1.921mg/mL)	3.239	3.253	3.252	3.197	3.130	3.072	3.004	2.773	2.425	2.098	0.072	>1:512000
IgG^[2]^	0.076	0.061	0.062	0.061	0.073	0.059	0.058	0.064	0.056	0.070	0.066	<1:1000

^[1]^Titer is the highest dilution with S/B (Signal/Blank) >=2.1 and the OD450 of the blank is the average of two technical replicates^[2]^ Isotype IgG control

**Fig. 2:**
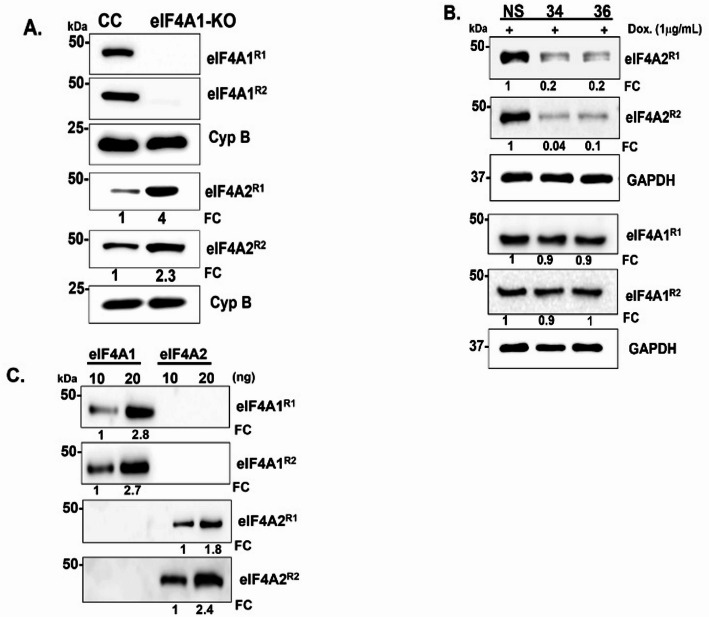
Validation of custom peptide-antibodies specific to eIF4A1 and eIF4A2. **A** Immunoblot analysis of CRISPR-control (CC) and eIF4A1 knockout (KO) cell lines using custom peptide antibodies against eIF4A1 and eIF4A2. R1 - Rabbit 1 and R2- Rabbit 2. Bands were normalized to cyclophilin B (Cyp. B). **B** Specificity assessment of eIF4A1 and eIF4A2 antibodies in doxycycline-inducible eIF4A2 knockdown cells. Two independent clones (34 and 36) and a non-silencing control (NS) were analyzed. Cells were treated with 1 µg/mL doxycycline to induce knockdown. Band intensities were quantified by densitometry and normalized to GAPDH. **C** Validation of antibody specificity using recombinant eIF4A1 and eIF4A2 proteins at 10 and 20 ng concentrations. All membranes were cut at the relative molecular weight (47 kDa) of eIF4A1 and eIF4A2, and the loading control for GAPDH and Cyp. B were also cut for loading control

Next, we checked the authenticity of the anti-ASQDSRSRDN (eIF4A1) antibodies by testing them on the lysates from the CRISPR-control and eIF4A1-KO MDA-Bone-Un cells.

that were previously generated [19]. The anti-eIF4A1 antibodies detected a distinct eIF4A1 band at the intended M_r_ range (47 kDa) in CRISPR-control but not in the KO lane, thus validating the anti-ASQDSRSRDN (eIF4A1) antibody (Fig. 2A). Additionally, the anti-eIF4A2 peptide antibody detected a clear upregulation of eIF4A2, further confirming its compensatory increase following genetic ablation of eIF4A1. Next, we checked the authenticity of the anti-GSADYNREHGC (eIF4A2) antibodies by testing them on the doxycycline-induced non-silencing control and shRNA-based knockdown (KD) lysates. Following the addition of doxycycline at 1 µg/mL, the anti-eIF4A2 antibodies followed the KD trend, thus validating the authenticity of these antibodies (Fig. 2B). Notably, we confirmed that the anti-eIF4A1 antibody did not cross-react with eIF4A2 in the KD lysates. Next, the peptide antibodies were tested against commercial, purified, recombinant eIF4A1 and eIF4A2 proteins. Both anti-eIF4A1 and anti-eIF4A2 peptide antibodies were specific and had no detectable cross-reactivity (Fig. 2C). Collectively, these results demonstrate that the affinity-purified anti-peptide antibodies generated are highly specific and reliable reagents for distinguishing eIF4A1 and eIF4A2.

#### Validation of commercially available antibodies

**Fig. 3:**
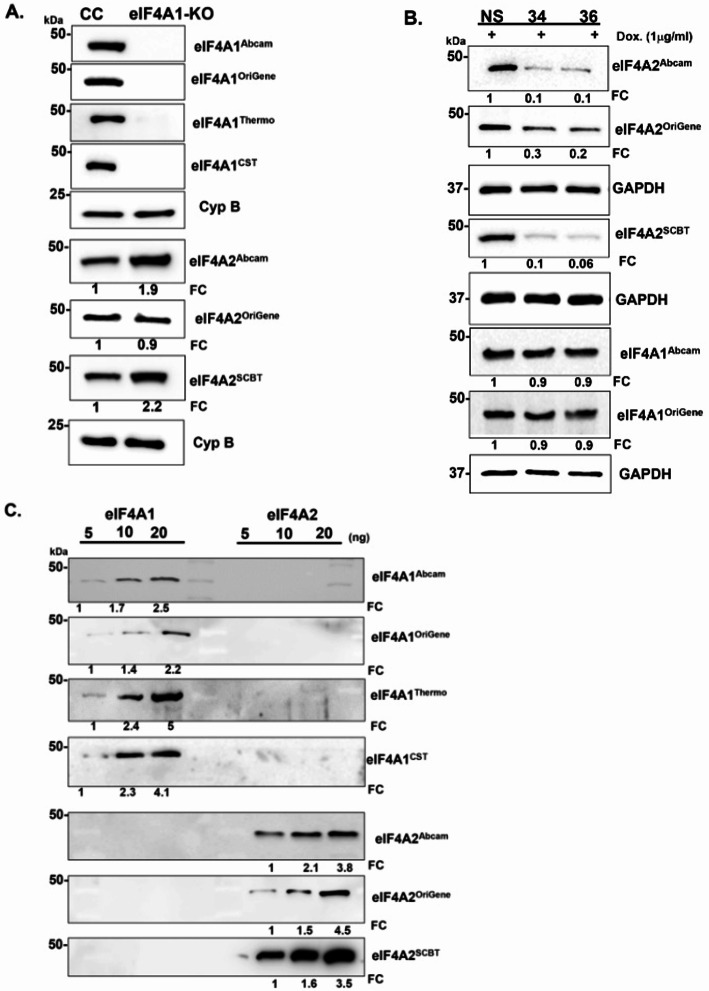
Validation of eIF4A1 and eIF4A2 expression using commercially available antibodies. **A** Immunoblot analysis of eIF4A1 knockout control (CC) and (eIF4A1-KO) cells using different commercially available eIF4A1 antibodies (Abcam, Origene Technologies, Thermo Fisher Scientific, and Cell Signaling Technology).Cyclophilin B (Cyp. B) was used as a loading control and relative band intensities are indicated. **B** Confirmation of eIF4A2 knockdown in non-silencing control (NS) and doxycycline-inducible eIF4A2 shRNA clones (34 and 36) using eIF4A1 antibodies from Abcam and Origene Technologies. **C** Immunoblot validation of recombinant eIF4A1 and eIF4A2 proteins using increasing amounts (5, 10, and 20 ng) of purified proteins. Various eIF4A1 antibodies (Abcam, Origene Technologies, Thermo Fisher Scientific, and Cell Signaling Technology) and eIF4A2 antibodies (Abcam, Origene Technologies, and Santa Cruz Biotechnology) were tested to confirm specificity and detection sensitivity. Relative band intensities are indicated. Under the current validation conditions, none of the tested commercial antibodies displayed detectable cross-reactivity between eIF4A1 and eIF4A2

Next, the commercial anti-eIF4A1 and anti-eIF4A2 antibodies were validated using the eIF4A1 KO (Fig. 3A) and eIF4A2 KD system (Fig. 3B), as well as by employing recombinant proteins (Fig. 3C).

In our previous study, a residual band was detected in eIF4A1-KO lysates when probed with the Cell Signaling anti-eIF4A antibody (#2013), suggesting possible cross-reactivity. This observation prompted us to generate custom peptide antibodies to ensure paralog-specific recognition. In the current study, we compared our peptide antibodies with additional commercial antibodies from Abcam, ThermoFisher, Cell Signaling and Origene Tech. detected eIF4A1 specifically and robustly in immunoblots and did not cross-react with eIF4A2. The anti-eIF4A2 antibodies from Santa Cruz and Abcam detected eIF4A2 specifically and did not cross-react with eIF4A1. The eIF4A2 antibody from Santa Cruz was more robust than the one from Abcam.

Our results unequivocally demonstrated that each antibody specifically recognized its intended target without cross-reacting with the other paralog. These findings confirm the specificity of these antibody tools, supporting their suitability for functional and mechanistic studies of eIF4A1 and eIF4A2 in cancer research.

#### Immunocytochemical validation of peptide-antibody specificity against eIF4A1 and eIF4A2

**Fig. 4:**
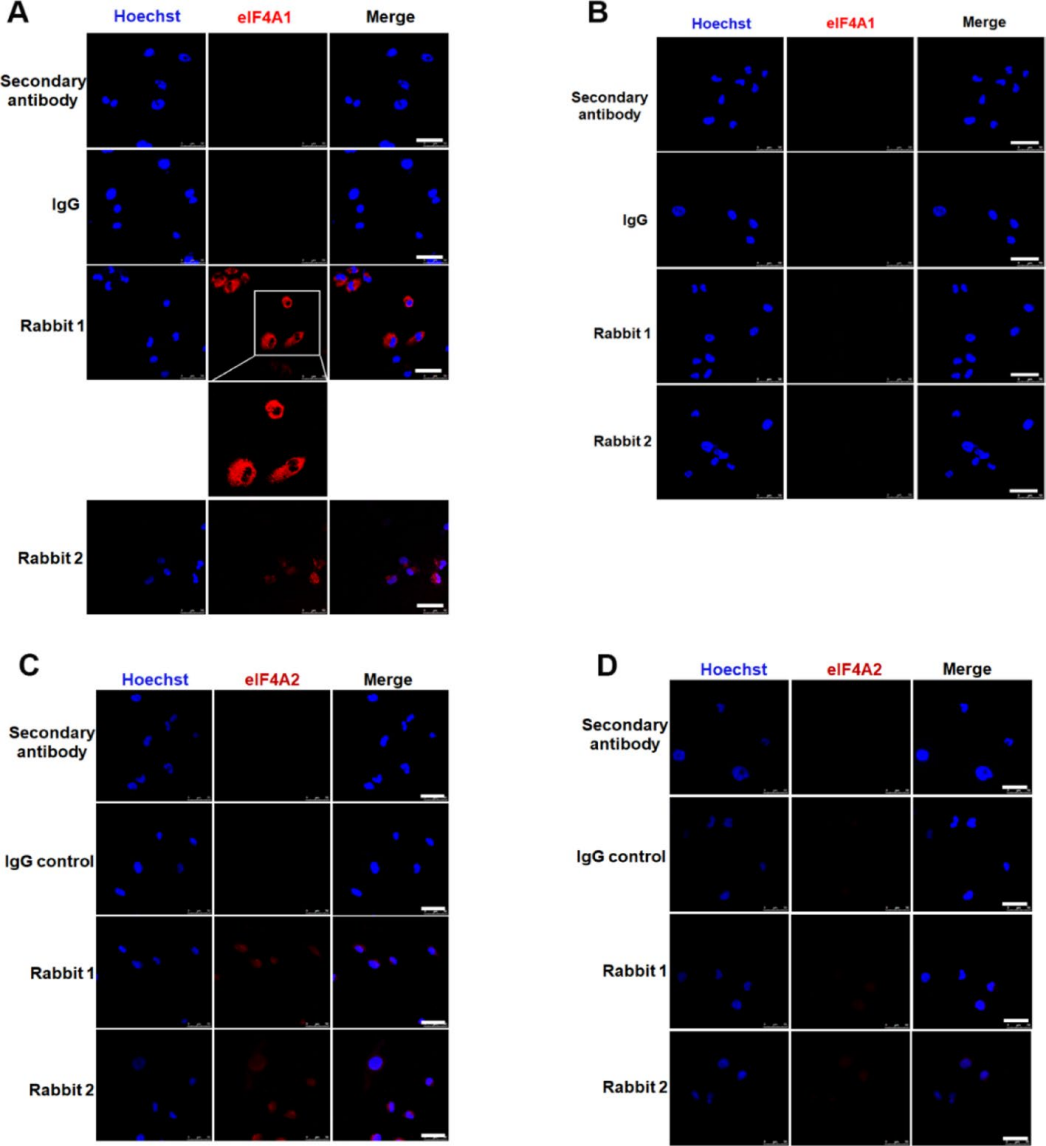
Peptide-antibodies against eIF4A1 demonstrate a high specificity and no cross-reactivity.** A–B** Both CRISPR-control and eIF4A1-KO cells were plated on collagen-coated coverslips and allowed to attach and spread overnight. They were then fixed and immunostained with two independent eIF4A1 peptide-antibodies (Rabbit 1 and Rabbit 2) or with control rabbit IgG and secondary antibody alone as negative controls. eIF4A1 and the nuclei were pseudo-colored red and blue respectively. Specific cytoplasmic distribution of eIF4A1 is detected in CC but totally absent in eIF4A1-KO cells, confirming antibody specificity. **C–D** Both control-silenced (NS) and eIF4A2 knockdown (eIF4A2-KD) cells were plated on collagen-coated coverslips and allowed to attach and spread overnight in complete DMEM media supplemented with puromycin and doxycycline (both at 1 µg/mL). They were then fixed and immunostained with two independent eIF4A2 peptide-antibodies (Rabbit 1 and Rabbit 2) or with control rabbit IgG and secondary antibody alone as negative controls. eIF4A2 and the nuclei were pseudo-colored red and blue respectively. A cytoplasmic and nuclear distribution for eIF4A2 is detected. Secondary-only and IgG controls show no detectable fluorescence, indicating minimal background or non-specific staining. Images represent single z-stack section acquired by Leica confocal microscopy of 1 μm optical slices, 63x magnification, scale bar = 50 μm. All images were acquired under identical confocal settings

To further assess the specificity of the custom peptide-antibodies, we performed immunocytochemistry (ICC) on eIF4A1 CC and KO cells using both Rabbit 1 and Rabbit 2 (Fig. 4A and B) anti-eIF4A1 antibodies from both rabbits revealed cytoplasmic punctate staining pattern, consistent with the localization of eIF4A1. The intensity of staining differed between the two antibodies: Rabbit 1 exhibited a stronger punctate signal compared to Rabbit 2, which displayed weaker fluorescence. This difference likely reflects the strength of host reactivity to the peptide antigen. The host rabbits employed to raise anti-eIF4A1 were administered an extra booster due to low initial ELISA titer.

Control samples treated with secondary antibody or with rabbit IgG showed no detectable fluorescence. However, ICC did not show intensity in eIF4A1-KO thus validating the specificity of the anti-eIF4A1 antibodies. Similarly, we validated the specificity of the anti-eIF4A2 peptide antibodies in our doxycycline-inducible eIF4A2 KD system. Compared to eIF4A1, eIF4A2 signal appeared weaker (Fig. 4C) as it is expressed at comparatively low level than eIF4A1. Both Rabbit 1 and Rabbit 2 antibodies displayed distinct fluorescence in NS control cells that were markedly reduced in KD clones (Fig. 4D), consistent with the KD efficiency confirmed by immunoblotting. Both NS and KD cells exhibited nuclear and cytoplasmic staining patterns which were absent in the primary and secondary antibody controls.

## Discussion

The development of paralog-specific antibodies for eIF4A1 and eIF4A2 represents a significant advancement in studying their distinct roles in translational control of many hallmarks in cancer biology. Given the high sequence similarity (~ 90%) between these paralogs [34, 35], generating antibodies capable of distinguishing between them posed a significant technical challenge. Our study is the first one to successfully validate the specificity of custom-generated and commercially available antibodies, thus confirming their ability to selectively recognize eIF4A1 and eIF4A2 without any cross-reactivity.

A key observation motivating this work was the detection of a residual band in eIF4A1-KO lysates when probed with the Cell Signaling Technology eIF4A antibody (#2013) [19]. This indicated potential cross-reactivity, likely due to sequence overlap between the eIF4A paralogs. Such findings underscore the necessity of rigorous antibody generation and validation using genetic models rather than relying solely on vendor-declared specificity. Consequently, we designed and generated our own paralog-specific peptide antibodies to clearly distinguish eIF4A1 from eIF4A2.

Using unique N terminal peptide epitopes of eIF4A1 and eIF4A2, we generated affinity purified rabbit polyclonal antibodies that demonstrated exquisite specificity in multiple validation systems. Immunoblot analysis demonstrated that each paralog-antibody detects its respective paralog with high specificity, as evidenced by the presence of distinct bands at the expected molecular mass (~ 47 kDa). The loss of eIF4A1 expression in KO cells and the reduction of eIF4A2 upon KD confirmed target specificity. When benchmarked against other commercial antibodies including those from Abcam, Origene Technologies, Thermo Fisher Scientific, and Santa Cruz Biotechnology, our custom antibodies displayed paralog sensitivity. These findings establish a foundation for leveraging these reagents in dissecting paralog-specific functions in translational control, cancer progression, and metastasis.

Our ICC results provided additional confirmation of antibody specificity and insight into the subcellular localization of eIF4A1 and eIF4A2. Both custom anti-eIF4A1 antibodies revealed a distinct cytoplasmic punctate staining pattern in the CC cells, consistent with previously reported localization of eIF4A1 [36]. No detectable fluorescence was observed in eIF4A1-KO cells, confirming the absence of non-specific binding. Interestingly, in the doxycycline-inducible NS control cells, anti-eIF4A2 antibodies produced relatively low fluorescence intensity, consistent with our immunoblot findings that elevated endogenous eIF4A1 expression corresponds to reduced eIF4A2 levels. This inverse relationship suggests a potential regulatory interplay between the two paralogs. However, previous studies have shown that increased eIF4A2 expression does not functionally compensate for the loss of eIF4A1 [24, 25]. In contrast, the anti-eIF4A2 antibodies displayed both nuclear and cytoplasmic localization in doxycycline-inducible NS and KD cells, with a punctate staining pattern similar to prior evidence reported in HeLa cells, where eIF4A2 has also been shown to exhibit punctate staining pattern both in the nucleus and cytoplasm [37]. The functional significance of this nuclear localization of eIF4A2 remains unclear and warrants future investigation.

The biological implications of these findings extend beyond antibody development. eIF4A1 has been established as an important regulator of oncogenic translation [17, 38, 39]. Previous studies have demonstrated that eIF4A1 is essential for the translation of mRNAs involved in cell proliferation, metastasis, and chemoresistance, particularly in TNBC [19, 20]. The antibodies generated and validated in this study may be useful tools for future studies aimed at understanding paralog-specific functions of eIF4A1 and eIF4A2 under physiological and pathological conditions. Conversely, eIF4A2 has been implicated in translational repression and microRNA-mediated gene silencing, indicating a potential tumor-suppressive role in specific contexts [4]. The ability to selectively detect eIF4A2 will facilitate further exploration of its novel functions in addition to microRNA-based repression. The robustness of these antibodies also opens new avenues for biomarker discovery and clinical applications. As eIF4A1 has been identified as a potential prognostic marker in various cancers [38, 40, 41], these antibodies could be employed in immunohistochemical analyses of patient tissues to assess eIF4A1 expression patterns in tumor progression and treatment response. Additionally, the ability to differentiate between eIF4A1 and eIF4A2 in tumor versus normal tissues may yield novel insights into their distinct roles in cancer biology. The availability of paralog-specific antibodies offers an opportunity to investigate differential expression patterns, subcellular localization, and functional redundancies or distinctions between these helicases in in vitro and immunohistochemical studies. These findings could aid in the development of more selective therapeutic strategies aimed at targeting eIF4A1-dependent oncogenic translation without disrupting the physiological functions of eIF4A2.

In conclusion, this study provides a fundamental resource for the cancer research community by finding highly specific antibodies to study eIF4A1 and eIF4A2 at the protein level. By enabling precise functional characterization of these paralogs, these antibodies will drive future research into translational control mechanisms, contribute to the identification of novel therapeutic targets, and ultimately support the development of more effective treatments. Moving forward, their application in mechanistic studies, drug screening, and patient stratification will be instrumental in advancing our understanding of eIF4A paralogs.

## Future perspectives

Several promising avenues emerge from this study. Future work should focus on the application of these antibodies in various experimental models, including immunohistochemistry to explore the spatial and temporal distribution of eIF4A paralogs in normal and cancer tissues. Additionally, quantitative proteomics approaches combined with these antibodies could provide deeper insights into paralog-specific interactomes and regulatory networks.

In TNBC, where eIF4A1 is known to drive oncogenic translation, future research that investigates the functional consequences of eIF4A1 following the depletion of eIF4A1, the loss of total eIF4A1 antibodies can be tracked using these antibodies. Understanding how eIF4A2 compensates for eIF4A1 loss or fails to do so, could reveal novel therapeutic vulnerabilities. Moreover, since small-molecule inhibitors lack paralog selectivity, these antibodies could be used to evaluate the effects of targeted inhibition on eIF4A1- versus eIF4A2-mediated translation, guiding the development of next-generation of paralog-selective inhibitors.

Finally, the role of eIF4A2 in translational repression further be expanded using these antibodies. Given its reported involvement in microRNA-mediated gene silencing, future studies should examine how eIF4A2 modulates the expression of tumor suppressors or oncogenes under different stress conditions. This may ultimately contribute to a more comprehensive understanding of translational control mechanisms and their implications in tumorigenesis.

## Supplementary Information

Supplementary Material 1

## Data Availability

The datasets generated and analyzed during this study are available from the corresponding author upon reasonable request. The ELISA data, antibody titer information, recombinant protein information and commercial antibody catalog numbers used in the study are detailed in the Methods and Results section. No publicly archived datasets were generated as part of this study.
