# Supplementary material for: Peptide antibody tools to dissect specific functions of eIF4A paralogs in cancer
Source: Discov Oncol. 2025 Nov 26;16:2309. doi: 10.1007/s12672-025-04155-x (PMC12748412; doi:10.1007/s12672-025-04155-x)
Supplement: Supplementary file 1 — Supplementary Material 1 [file 12672_2025_4155_MOESM1_ESM.pdf]

Peptide Antibody Tools to Dissect Specific Functions of eIF4A Isoforms in Cancer

Shobhit Srivastava[1], Azeezat Osikoya[1], David Terrero[1]and Dayanidhi Raman[1]

[1]Department of Cell and Cancer Biology, University of Toledo Health Science Campus, Toledo, OH, United States of America

Supplementary figures

Fig. 2A

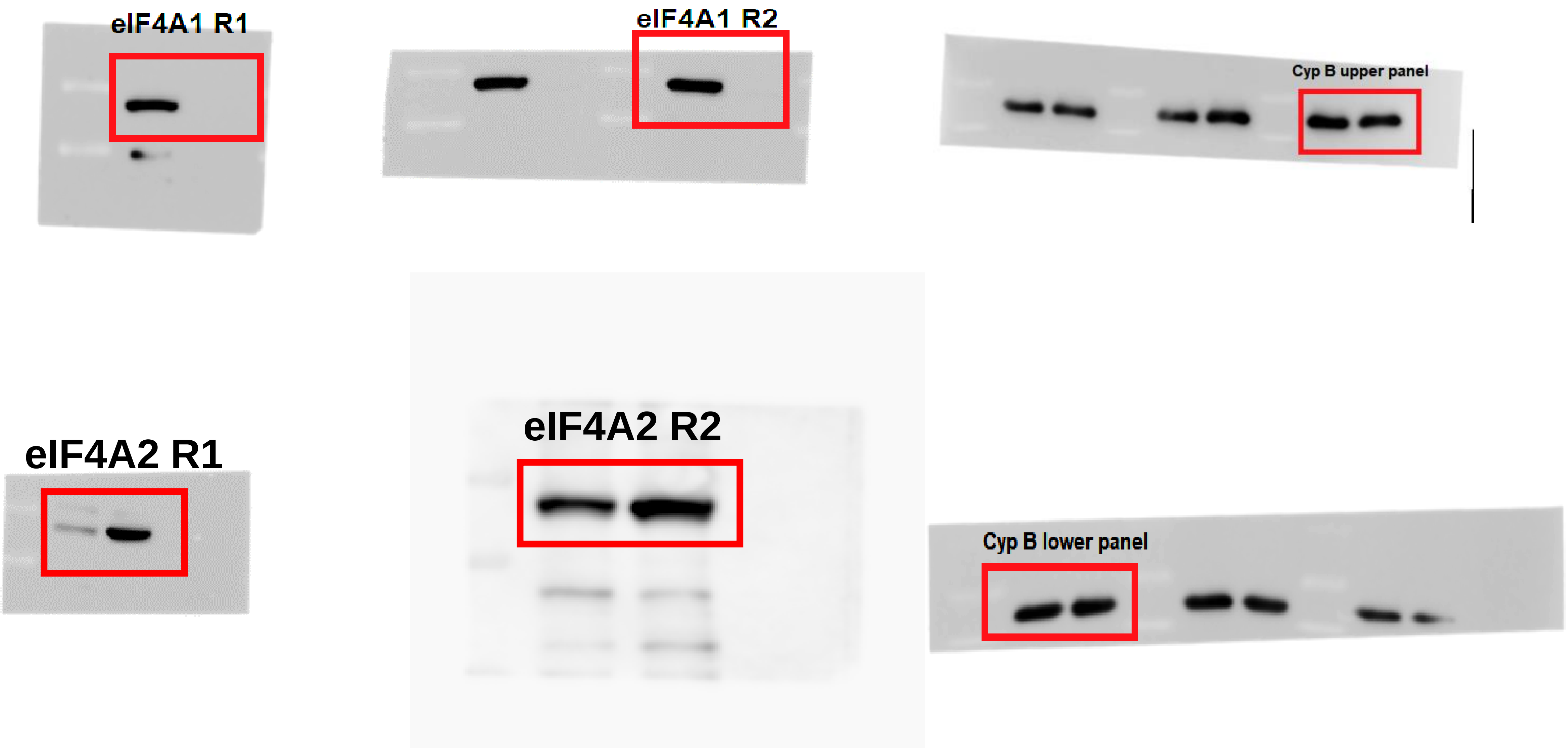

Membranes are cut near eIF4A1 and eIF4A2 molecular weight (47kDa) and Cyp B (25 kDa). Images shown here have the protein marker visible.

Fig. 2B

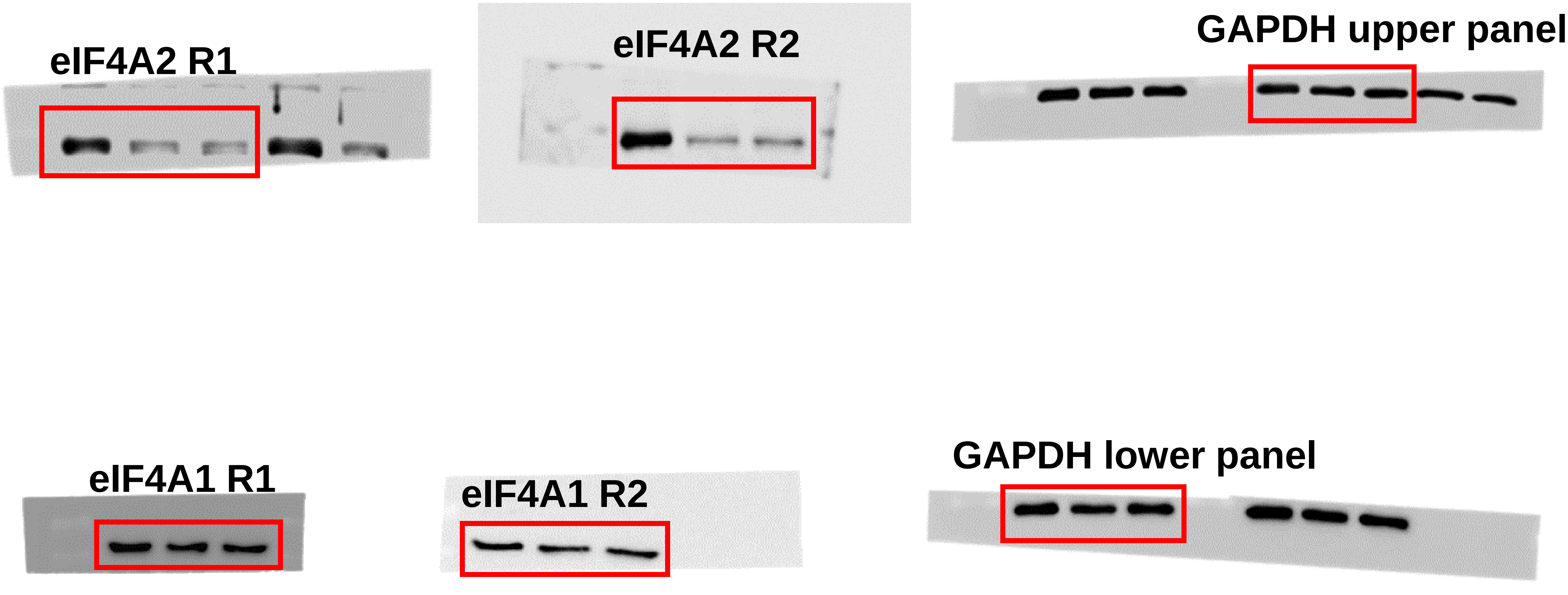

Membranes are cut near eIF4A1 and eIF4A2 molecular weight (47kDa) and GAPDH (37kDa).
Images shown here have the protein marker.

Fig. 2C

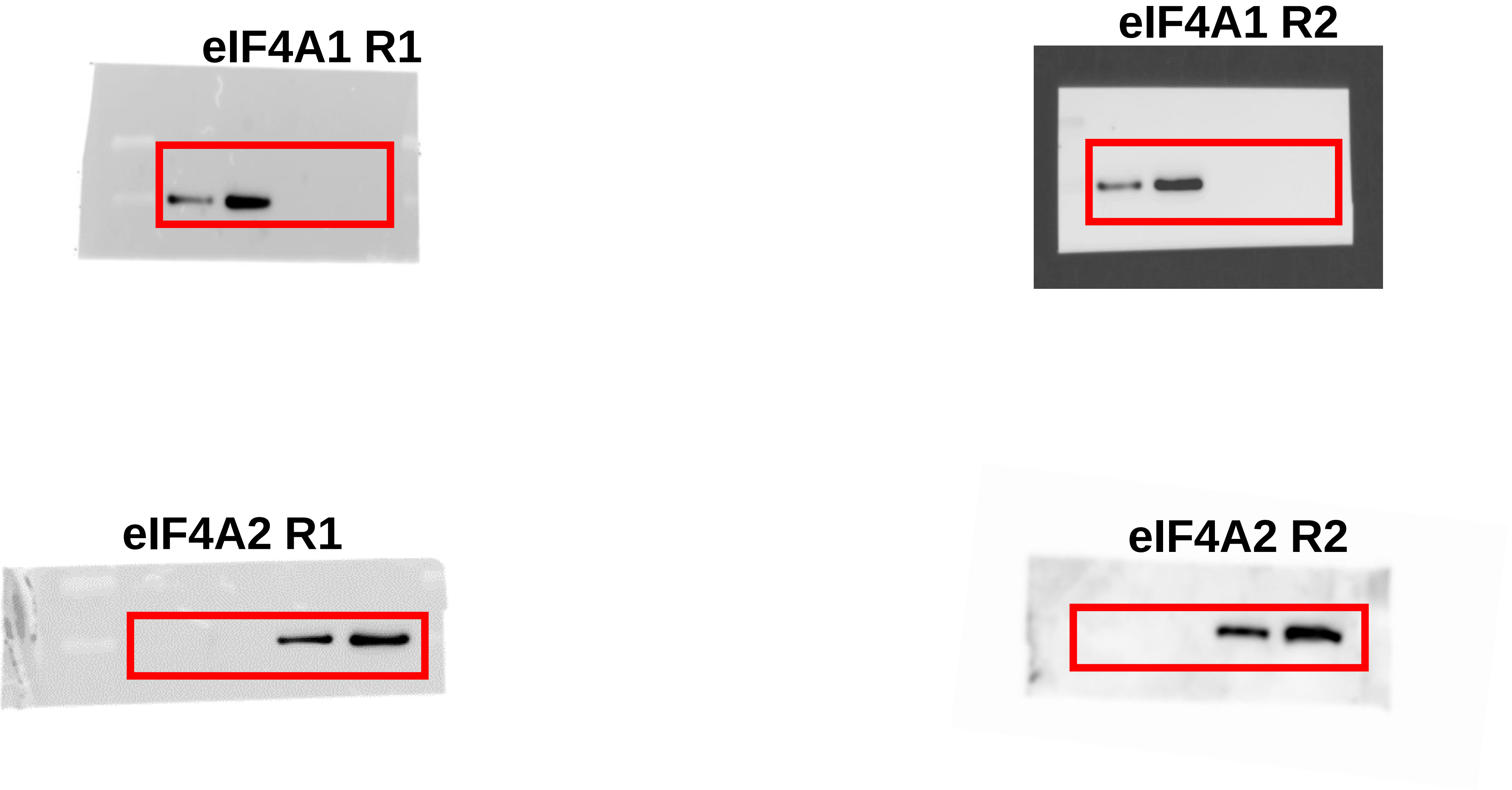

Membranes are cut near eIF4A1 and eIF4A2 molecular weight (47kDa). Images shown here have the protein marker visible.

Fig. 3A

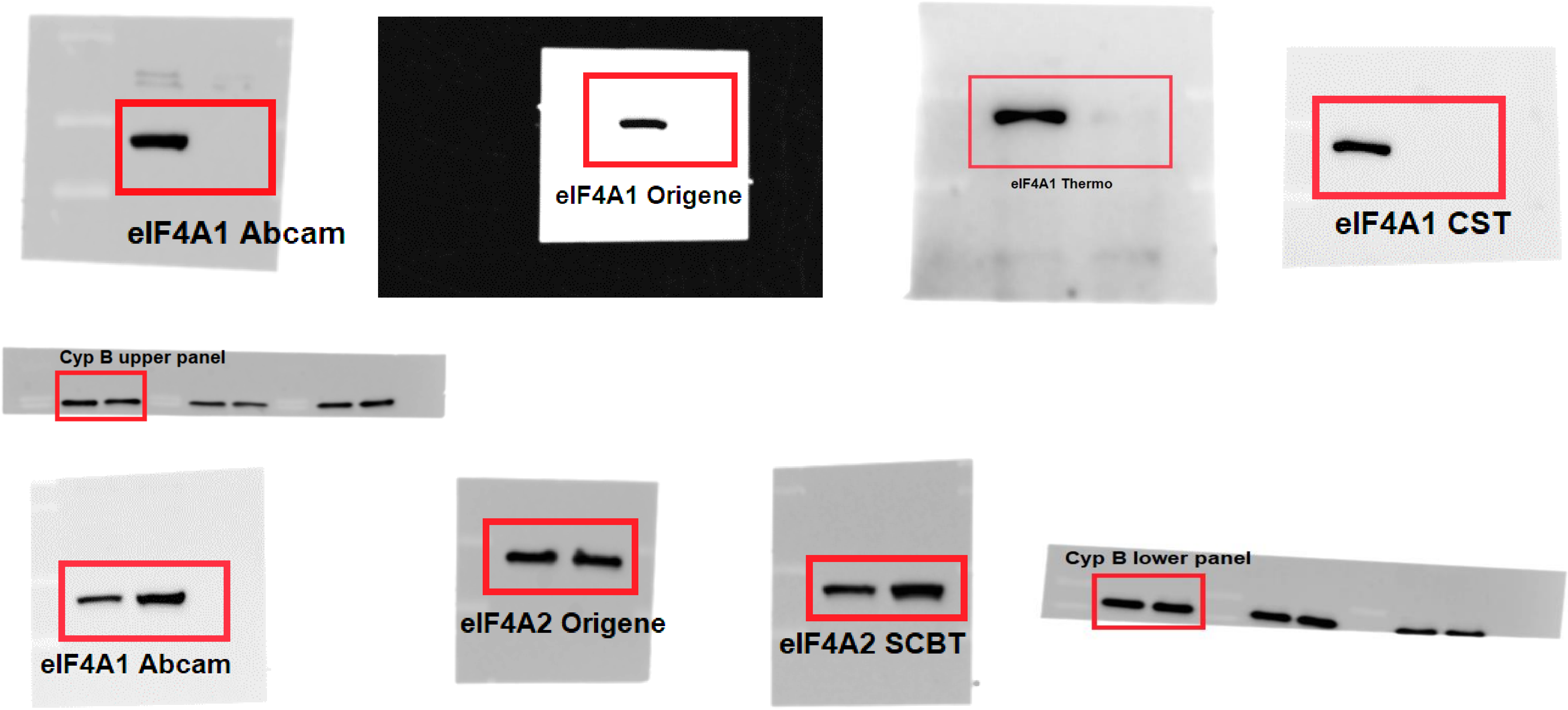

Membranes are cut near eIF4A1 and eIF4A2 molecular weight (47kDa) and Cyp B (25kDa). Images shown here have the protein marker visible.

Fig. 3B

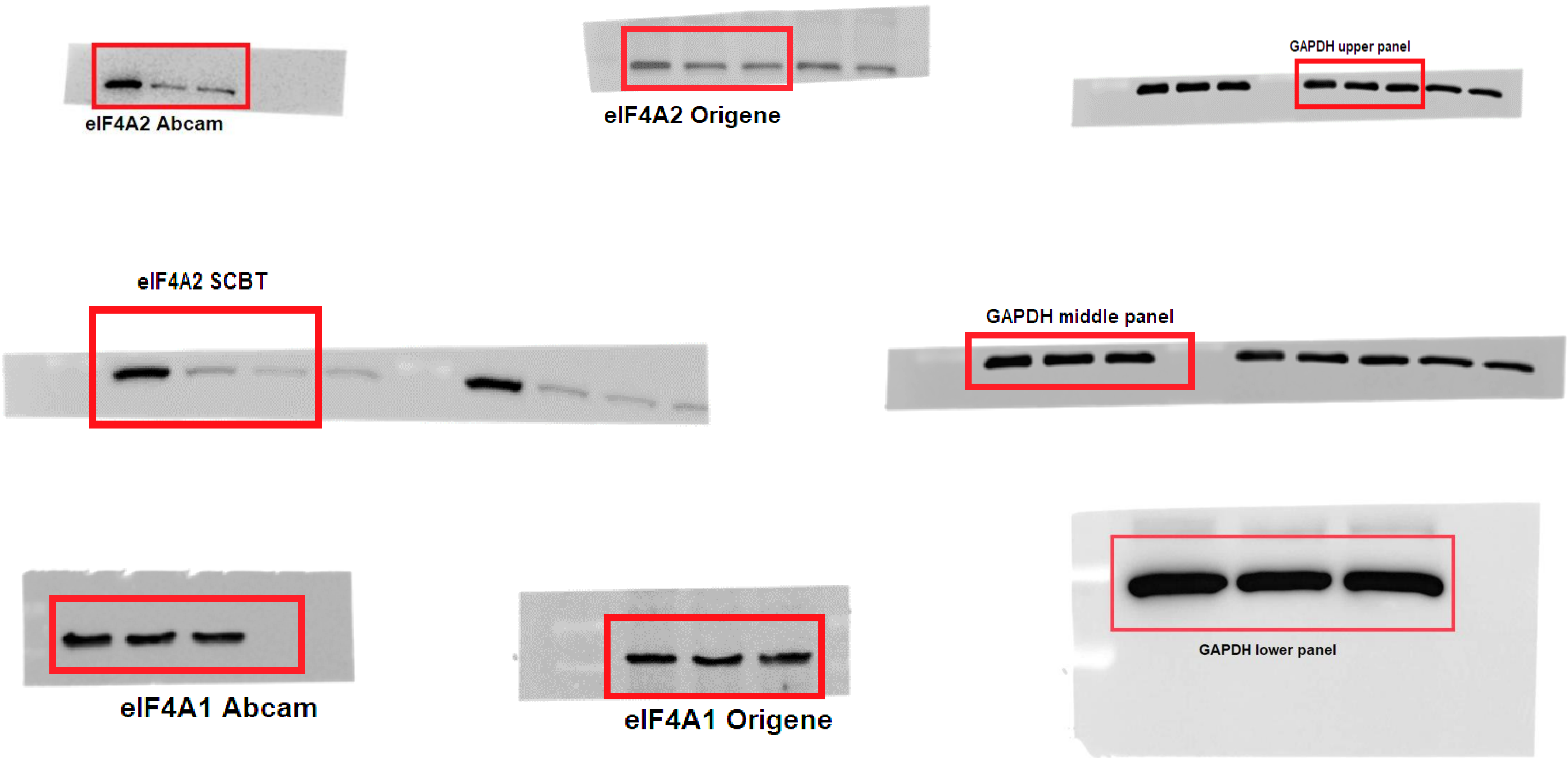

Membranes are cut near eIF4A1 and eIF4A2 molecular weight (47kDa) and GAPDH (37kDa). Images shown here have the protein marker visible.

Fig. 3C

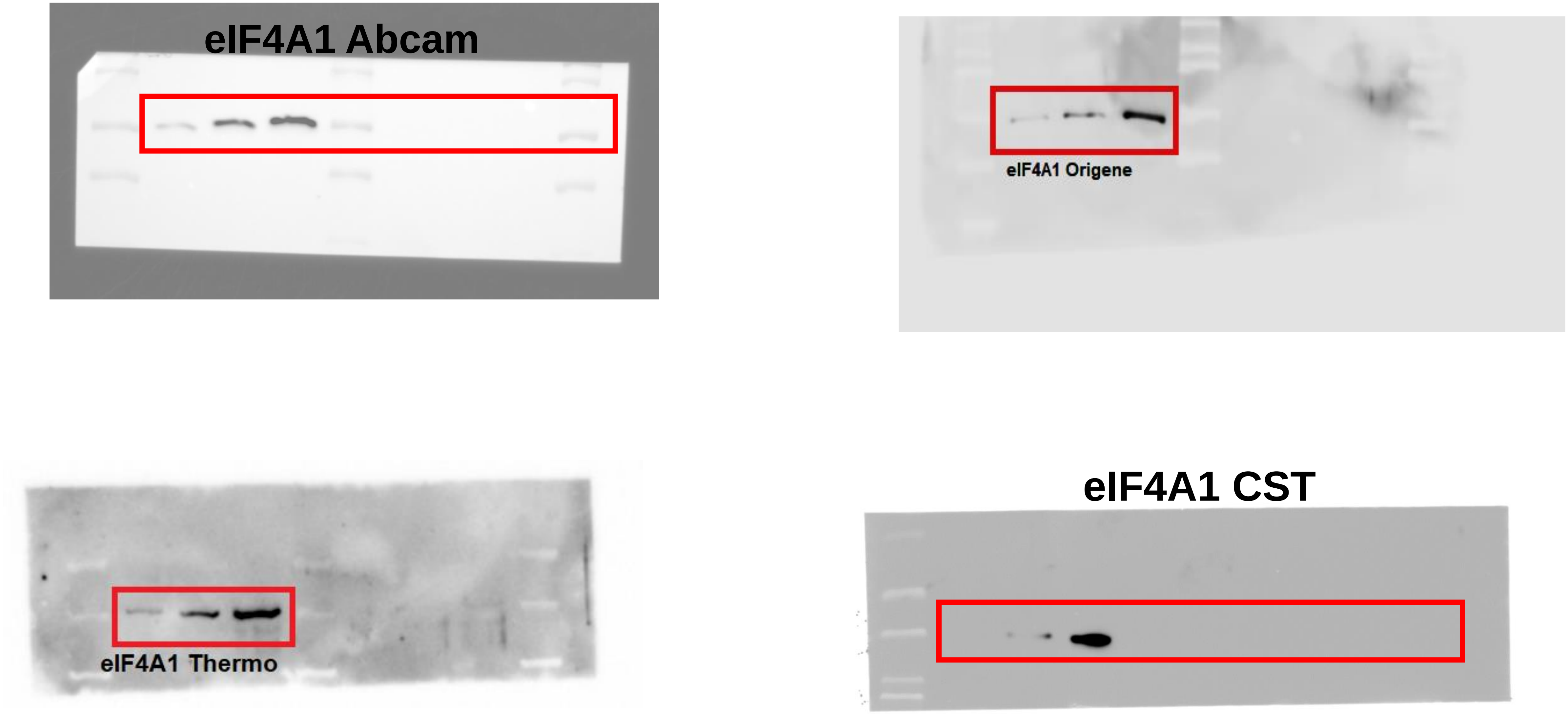

Membranes are cut near eIF4A1 molecular weight (47kDa). Images shown here have the protein marker visible.

Fig. 3C

eIF4A2 Abcam

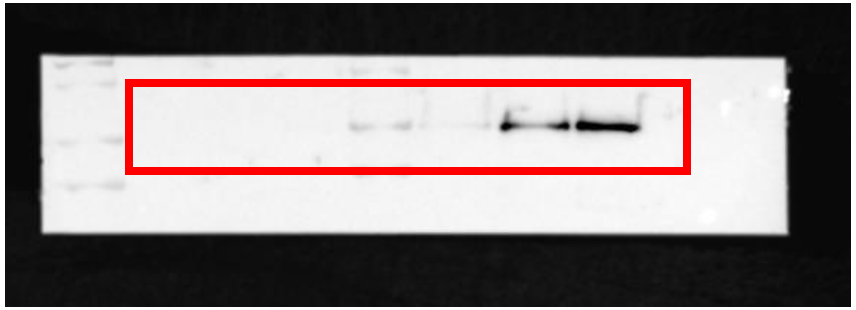

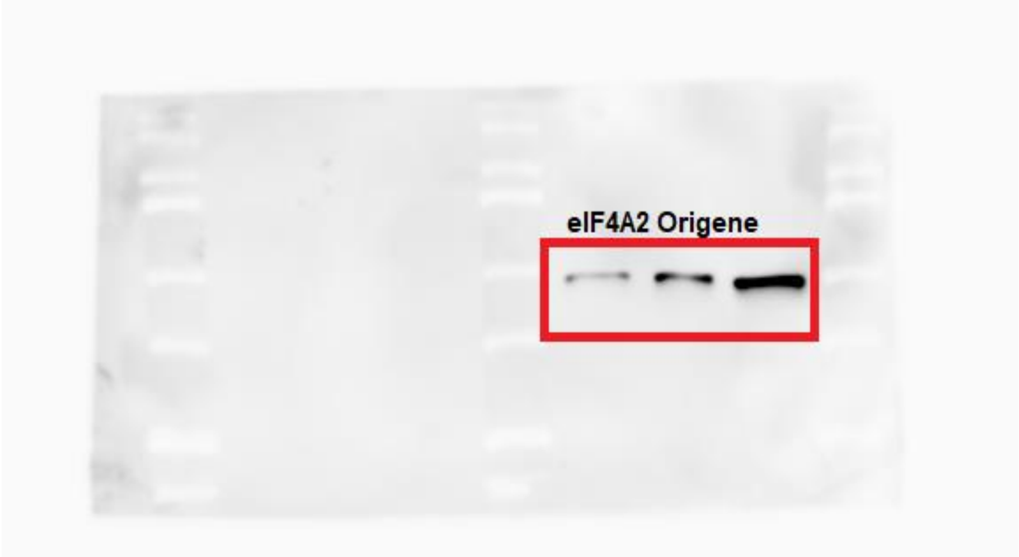

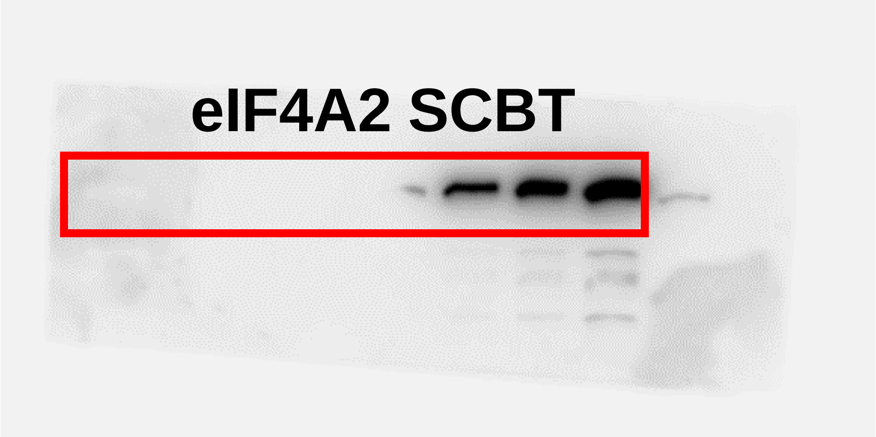

Membranes are cut near eIF4A2 molecular weight (47kDa) and Cyp B (25kDa). Images shown here have the protein marker visible.
